# Preventing Child Drowning in Indonesia: A Community-Informed Health Promotion Perspective

**DOI:** 10.1177/10901981251330487

**Published:** 2025-04-23

**Authors:** Muthia Cenderadewi, Richard C. Franklin, Prima B. Fathana, Susan G. Devine

**Affiliations:** 1 James Cook University, Townsville, Queensland, Australia; 2 University of Mataram, West Nusa Tenggara, Indonesia; 3 Royal Life Saving Society–Australia, Sydney, New South Wales, Australia

**Keywords:** drowning, health behavior, Health Belief Model, health education, health promotion, injury prevention, low- and middle-income countries

## Abstract

Child drowning is a significant public health issue in Indonesia; however, there remains a lack of understanding within communities of the risks and how to prevent it. This qualitative study aimed to explore existing and suggested actions undertaken by parents and communities to prevent child drowning. Seven focus group discussions were conducted, comprising 62 participants, with parents of children below 5 years and village community leaders from seven villages on Lombok Island, West Nusa Tenggara, Indonesia. Participants were recruited with purposive and snowball sampling methods. The thematic analysis used both deductive, applying the Health Belief Model and the Health Promotion Framework, and inductive approaches. The results highlighted the focus that participants placed on individual-focused, behavioral drowning interventions, particularly through swimming lessons for school-age children and educational programs on life-saving skills for parents and community members. While participants acknowledged the importance of midstream interventions, such as safety measures around water bodies and community-based safe places for children, alongside population-based upstream interventions such as advocating for policies, regulations, and intergovernmental agency collaboration, there was limited understanding on the roles of the education and health departments in preventing child drowning. Participants reported inconsistent and insufficient implementation of swimming lessons in schools. Further research into formal integration of swimming training into school curricula and its impact on reducing child drowning rates, development of contextually relevant water safety promotion approaches, and alignment of cross-sector partnerships is imperative to ensure effective and sustainable drowning prevention efforts in Indonesia.

Child drowning remains a significant public health issue worldwide, particularly across low- and middle-income countries (LMIC), including in Indonesia (Franklin et al., 2020; World Health Organization [WHO], 2021; World Health Organization Regional Office for the South-East Asia, 2021). Between 2005 and 2019, Indonesia consistently witnessed high drowning mortality fatalities among children below 5 years of age, averaging 9.7 deaths per 100,000 annually (Cenderadewi et al., 2024), exceeding global rates (5.5 per 100,000 in 2019) (Tan et al., 2023) and those in high-income countries, such as Australia (1.1 per 100,000 in 2022) (Royal Live Saving–Australia, 2023) and the United States (3.1 per 100,000 in 2022) (Clemens et al., 2023), for the same age group. Children below 5 years of age residing in the eastern part of Indonesia, including in Papua, Maluku, and Nusa Tenggara, recorded the highest fatalities from drowning (Cenderadewi et al., 2024).

Located in Southeast Asia, Indonesia has the world’s fourth largest population of more than 270,200,000 people (Indonesian National Bureau of Statistics, 2021). It is the world’s largest archipelagic state consisting of 17,500 islands, and faces frequent hydrometeorological disasters, increasing the risk of drowning for children (Indonesian National Bureau of Statistics, 2021). However, there is limited information on local community perceptions and practices regarding child drowning prevention in Indonesia (Cenderadewi et al., 2023). Understanding these perceptions and current practices is crucial for developing effective, context-specific drowning prevention strategies.

Childhood drowning is a complex issue requiring a multi-strategic approach that addresses behavioral, environmental, health and social factors (Cenderadewi et al., 2020; Franklin et al., 2017; Guevarra et al., 2021; Peden & Franklin, 2020). A comprehensive framework such as the Health Promotion Framework (HPF) can assist in mapping current approaches being used to address childhood drowning, as well as identifying gaps and community-recommended approaches (Talbot & Verrinder, 2017). The HPF is grounded in the inter-related socio-ecological determinants of health, encompassing: (1) biomedical approaches for individual risk assessment, (2) behavioral approaches to develop awareness and skills, and (3) socio-ecological approaches for community action and policy development (Talbot & Verrinder, 2017).

This study used both the HPF and the Health Belief Model (HBM) to complement each other, providing a comprehensive understanding of childhood drowning from both a multi-level health promotion perspective and an individual behavior standpoint. The HPF examined the broader context of drowning prevention through individual, community, and policy levels, whereas the HBM focused on personal beliefs and motivations influencing health behaviors. The HBM posits that health behavior is driven by perceptions of susceptibility, severity, benefits, barriers, and ability to act (self-efficacy) (Champion & Skinner, 2008; Etheridge et al., 2023). By utilizing both models, the study aims to explore both socio-ecological factors and personal, attitudinal barriers to water safety, informing targeted interventions in Indonesia.

## Research Aims

This qualitative study aimed to explore the existing and suggested actions undertaken by parents and communities to prevent child drowning.

## Research Questions

This study answered the following questions:

***Research Question 1:*** What are the current practices among Indonesian parents and communities for preventing child drowning in their community?
***Research Question 2:*** What strategies do Indonesian parents and communities recommend for preventing child drowning in their community?

## Method

### Study Design

This qualitative study is a component of a larger mixed-methods inquiry into fatal unintentional drowning in Indonesia, comprising three phases: (1) a scoping review (Cenderadewi et al., 2023), (2) a population-based retrospective cohort study (Cenderadewi et al., 2024), and (3) the qualitative investigation reported here. The selection of a qualitative design aimed to expand on the findings of the quantitative study, which identified high mortality rates among children aged below 5 years, particularly in eastern Indonesia including in Nusa Tenggara (Cenderadewi et al., 2024). The exploratory qualitative approach (Creswell, 2014; Hunter et al., 2019) enabled exploration into participants’ perceptions and experiences in identifying and mitigating child drowning risks within Indonesian communities, an area underexplored in existing literature.

### Research Setting

This study was conducted in seven villages across all districts (West Lombok, North Lombok, East Lombok, Central Lombok, and Mataram) of Lombok Island, situated in the West Nusa Tenggara (WNT) province of Indonesia. The selection of WNT as the study location was based on its high below-5 drowning rates of 12.6/100,000 for males and 6.1/100,000 for females in 2019, one of the highest across all Indonesian provinces (Cenderadewi et al., 2024). WNT’s rural characteristics and status as one of Indonesia’s poorest health-performing provinces, underscored by its high below-5 mortality rate of 29 deaths per 1,000 live births in 2022, highlighted the significance of investigating the region, as it represents the high-risk populations of economically disadvantaged children living in rural areas of Indonesia (Indonesian, National, Bureau, of, & Statistics, 2023, 2024; United Nations Inter-Agency Group for Child Mortality Estimation, 2023).

### Sample Selection and Recruitment

Participants were eligible if they were parents of children aged 1 to 4 years and or identified as community leaders in villages located in coastal sub-districts or near inland water bodies on Lombok Island, WNT. This age group is particularly vulnerable to drowning, making parents valuable sources of information on preventive measures. Community leaders, including village chiefs, elders, religious figures, and community health workers, were selected for their knowledge of community norms and actions related to drowning prevention.

Face-to-face recruitment was conducted by research team members familiar with the local context to ensure cultural sensitivity. The process began with the researchers using purposive sampling to identify initial participants—village chiefs and community health workers—who then facilitated snowball sampling to identify key informants with relevant knowledge on child drowning who met inclusion criteria.

### Data Collection

Seven focus group discussions (FGDs) (*n* = 62) were conducted at various community locations and times of day between October 2023 and March 2024, until data saturation was achieved. This methodology captured individual stories and community practices on child drowning prevention, fostering deeper insights through iterative participant interactions (Kitzinger, 1995; Krueger & Casey, 2015). Written consent was obtained prior to the focus groups, and participant characteristics were collected using a demographic questionnaire. The moderator guide, guided by HBM (Champion & Skinner, 2008) and findings of a previous scoping review (Cenderadewi et al., 2023), was tested for face validity prior to implementation, resulting in minor modifications of the prompts (Appendix A).

FGDs, lasting 50 to 60 minutes, were facilitated by two female Indonesian researchers who are fluent in Indonesian and local Sasak language. The lead researcher facilitated discussions, whereas the second team member took notes and observed interactions. Parents and community leaders were included in the same FGDs to foster richer discussions around prevention strategies, as well as childrearing and water safety practices, at both household and community levels, enhancing the depth and dependability of findings. To achieve data saturation, the researchers continued data collection until redundancy in the information collected was reached, at which point new themes or insights ceased to emerge.

All focus groups were audiotaped with consent, transcribed verbatim, and translated into English by the lead researcher. Transcripts were back-translated by the other Indonesian team member and reviewed by two senior researchers to verify accuracy.

### Analysis

Translated transcripts, demographic information, and field notes were entered into NVivo Version 20. The thematic analysis (Clarke & Braun, 2013) used both deductive analysis using constructs of HBM (Champion & Skinner, 2008) and HPF (Talbot & Verrinder, 2017), and inductive approaches. The HPF provided a broad framework for understanding multi-level health promotion strategies. Although the HBM was not the primary focus of the analysis, it offered valuable insights into individual and community beliefs and decision-making processes that shape water safety behaviors.

After becoming familiar with the entire data set by reviewing the interview transcripts multiple times, the lead researcher independently coded the data. Coding and excerpts were then reviewed by senior researchers. The lead researcher and senior researchers iteratively reviewed each theme to accurately confirm the reflection of the data.

To ensure the dependability, conformability, transferability and authenticity of the study, several measures were undertaken: (1) Purposive sampling; (2) Extended engagement, involving ongoing interaction with the community before, during, and after data collection to acquire a thorough understanding of participants’ narratives; (3) Gathering of participant demographics; (4) Reflective note-taking and recording of observations; (5) Member checking, conducted in two phases: during each FGD to confirm key points with participants, and through follow-up interviews after transcribing the FGDs; (6) Validation of data accuracy through translation processes; (7) Examination of references, codes, and themes by multiple researchers to ensure data accuracy and consistency; (8) Consensus discussions on code definitions, coding of references, and themes; (9) Incorporation of verbatim quotations; and (10) Creation of an audit trail detailing data collection and analysis.

### Ethics Approval

Ethical approval was granted by the Human Research Ethics Committee (HREC) of the University of Mataram — Indonesia (Ethics Approval number 044/UN18.F8/ETIK/2024) and acknowledged by James Cook University’s HREC (External HREC Approval Acknowledgment reference number H9088).

## Results

Sixty-two participants participated in the FGDs, including mothers (53.2%, *n* = 33), fathers (9.7%, *n* = 6), and village community leaders (37.1%, *n* = 23). Most participants were female (75.8%, *n* = 47) and aged between 25 and 44 years (51.6%, *n* = 32). Most participants had not completed primary education (58.1%, *n* = 36) (Appendix B).

Data analysis revealed five key thematic areas related to the current strategies that were being undertaken to prevent drowning and potential prevention strategies suggested by participants: (1) Stakeholders in drowning prevention responsibility; (2) Health information and communication agents; (3) Community education and skills development; (4) Community action; (5) Policy development and enforcement. The findings are detailed below under the respective theme headings and synthesized in Table 1, which also features additional participant quotations for clarity.

**Table 1.** Definitions of Themes and Illustrative Quotes Supporting Interpretations of Themes.

Themes	Definition and inclusions	Subthemes	Representative excerpts
Stakeholders in drowning prevention responsibility	Participants’ views on stakeholders responsible for preventing drowning.	Multisectoral responsibilities	*“I think the most important thing is that the local government needs to reach the people with knowledge, the professionals, to provide education, information sessions for each community. Things we need to pay attention to, regarding the danger of drowning.”* (Group 7, female, participant ID number: G7F1) *“Maybe the Department of Tourism [part of the local and national government] could come here to inform us [on drowning prevention].”* (Group 7, female, participant ID number: G7F3) *“BASARNAS [the Indonesian National Search and Rescue Agency], yes, they are responsible [to prevent drowning]. If there are people drown on the beach, BASARNAS will search for them, right.”* (Group 3, female, participant ID number: G3F2)
		Parents	*“It’s the parents that are responsible [to prevent drowning]. Both parents. Some children fear their mother more, but some fear their father more. It should be the responsibility of both parents.”* (Group 5, female, participant ID number: G5F2) *“For me, personally, it’s fathers’ responsibility [to prevent drowning in children]. Fathers are most responsible for ensuring the safety of the children. Fathers have more authority. Mothers, we spend so much time with our children that they no longer listen to us. But once the father speaks, it is different. Children will listen to their father more.”* (Group 5, female, participant ID number: G5F6)
Health information and communication agents	Participants’ views and practices on prevention strategies which promote attitudes, beliefs, and behaviors that prevent drowning. This includes parenting or family-focused prevention strategies to promote water safety-promoting norms and behaviors in the family.	Government	*“There must be socialisation, information, education sessions, provided by the local government. . . . For us, new parents, for example, it is confusing if we are to experience it [child drowning]. We need to be educated on how to respond when things like drowning happen to our children. That is the most important thing for us. First, education needs to be provided.”* (Group 6, male, participant ID number: G6M1) *“Even on the tsunami, no one came to explain things to us. We only had our information from social media. Social media helps, but I think we still need instructions on this [preventing drowning] by the village apparatus, because not everyone is using social media.”* (Group 5, female, participant ID number: G5F6)
		Parents	*“We tell them [children] that when it rains, don’t play at the ditches.”* (Group 1, male, participant ID number: G1M3) *“Children are curious. Even if they’re in the swimming pools with their parents, for example, pools have different level of depths, and children can just run and plunge into the deeper pool. So as parents you need to tell them what to do beforehand. You need to explain things to them. Otherwise, they’ll just jump into any water.”* (Group 4, female, participant ID number: G4F3)
		Media	*“There are more news reports on drowning these days, including from celebrities. Like, if any celebrity or their children drown, we then become more aware. The news becomes a lesson for us, that we need to be more cautious. We need to be careful, to not too easily trusting other people, including to let our children being supervised by other people.”* (Group 6, female, participant ID number: G6F1) *“Yes, we heard recent news on drowning cases on mass media, that is why we need to be more cautious. We need to supervise better, especially on children. During the rainy season, especially.”* (Group 5, male, participant ID number: G5M1)
Community education and skills development	Participants’ views and experience on specific skills training needed to empower individuals with the knowledge and abilities necessary to safely navigate aquatic environments and to assist others in distress.	Swimming lessons for school-age children	*"No children here have swimming lessons. They learn to swim by themselves. Sometimes the father could take them to the beach. I live near the beach, so the kids’ father takes my kids to the beach. There are swimming lessons provided by the school since primary school. Once every semester. As long as the child attends this, this grade taking for sport education, they will receive a grade. They will be marked, just by attending the session. We have to prepare their transport fee, and entrance fee for the swimming pool. So, the school usually will arrange a bemo [a small van used for public transport] and we pay for the cost.”* (Group 3, female, participant ID number: G3F5) *“There are swimming lessons provided by the school, but only for junior high school. About twice a year. Once every semester. They call it grading, grading the swimming ability and that’s it. But it is far to swim. My kids are in junior high school, but I still need to drop my kids at the swimming pool, because they cannot ride motorbike. Even the cost of having them go for swimming [lessons arranged by the school] twice a year is already difficult for me, let alone more often than twice a year. If it’s, say, once a month, oh no. No. If there is a pool around here to swim, the children would love it very much. They probably won’t leave the pool ever! As long as it has swimming instructors.”* (Group 2, male, participant ID number: G2M2)
		First aid training	*“We don’t know how to do that, providing first aid for drowning. We actually need that education. We could only scream for help. We don’t know how to help with drowning.”* (Group 6, female, participant ID number: G6F3) *“For us, for instance, we perhaps have only watched in on tv, how to save people who are drowned. But we don’t know how to do it. We need to be taught.”* (Group 6, female, participant ID number: G6F1)
Community action	Participants’ views and practices on community action for social and environmental changes to create safer environment for children.	Providing unsupervised safe places	*“Having a playground for the children perhaps can be done [to prevent child drowning]. A place where young kids could be gathered in one place. Especially for younger children. If we could not find our children at home, we only need to look there.”* (Group 2, male, participant ID number: G2M2) *“We don’t have any space for children to play around here. If there is a playground, a space to play around here, perhaps children will not go as far to play. There is no open space these days. It will be great if a playground can be provided for the children.”* (Group 3, female, participant ID number: G3F2)
		Providing supervised safe places	*“Actually, people here are willing to look after one another, to watch each other’s children. People in the village are mostly helpful to one another. If there is a facility, a childcare, in this village, to have the children being watched, that would be helpful to stop the children from playing in streams.”* (Group 6, female, participant ID number: G6F1) *“Such as having a preschool [in the community]. That would be a good idea to prevent child drowning.”* (Group 4, female, participant ID number: G4F3)
		Barriers	*“Yes, kids were curious about it [the uncovered well], what was in there, so they got closer to well, they peeked over the well. We were worried about the possibility of children taking a plunge into the well. So, most of us have covered our wells.”* (Group 2, female, participant ID number: G2F1) *“Ponds or streams are not being fenced. Never. The ditches now often are covered by concrete, like concrete slabs. Actually, not really [ditches being covered]. It is [covered] in front of people’s houses only, so it depends on the owner of the house.”* (Group 1, female, participant ID number: G1F5)
Policy development and enforcement	Participants’ views that look at policies and regulations that help create a climate in which water safety is encouraged or inhibited.	*“Bathing pools are not regulated. It should be regulated, that only children that come with their parents can enter, but it’s not regulated. So, any children can enter bathing pools any time they wish.” (Group 4, female, participant ID number: G4F3)* *“No one on board of boats use lifejackets. Never. The boat crews never let people [passengers] know that we must use lifejackets and where the lifejackets are stored. And I have taken a few boat and ferry rides, to Bali and Java, but there was no such information provided about lifejackets. There should be lifejackets provided, as required. Sometimes there were lifejackets around, but at other times, none were seen. No, there was no information on that, especially on smaller boats. No one tells you what to do.”* (Group 2, male, participant ID number: G2M1)	

### Theme 1—Stakeholders in Drowning Prevention Responsibility

Participants shared their perspectives on the stakeholders they felt were responsible for preventing drowning. Across all age groups, most participants viewed parents as the main actors responsible for preventing drowning in children.

Parents would be responsible [to prevent drowning]. It’s impossible to rely on the government for everything. It’s too far-fetched. (Group 4, female, participant ID number: G4F3)

Participants highlighted the shared responsibility of government sectors in preventing drowning. They emphasized the involvement of child protection services, rescue agencies, environmental sectors, the fire department, and the tourism department as key actors in child drowning prevention. However, nongovernmental stakeholders were not mentioned.

There must be socialisation, information, education session[s], provided by the local government, from relevant government stakeholders, such as the ones dealing with the environment and with child protection. . . . from child protection agency, SAR [Search and Rescue] team, firefighters. Those involve in rescue efforts. (Group 6, male, participant ID number: G6M1)

Meanwhile, only one participant cited the education sector’s responsibility in drowning prevention, stressing the need to include swimming lessons in school curriculum. No mention was made of the education department’s involvement in other prevention aspects or of the health department’s role in child drowning prevention.

I think the government is also responsible [for drowning prevention]. I think swimming lessons could be a part of the school’s curriculum. . . . it doesn’t need to be a total revision of the curriculum. This could just be a supplementary activity. (Group 7, female, participant ID number: G7F1)

### Theme 2—Health Information and Communication Agents

Participants discussed various health communication strategies focusing on fostering attitudes, beliefs, and behaviors to prevent drowning. These included family-focused approaches and government-led initiatives.

Most participants emphasized the need for government-supported health information on child drowning prevention. Some stressed the value of in-person sessions, especially for reaching communities with limited media access.

People need to be told what to do by the government, to be informed. Not all of us watch tv or get hold of handphone. If we meet like this [face-to-face], it is better for the mothers, to let the mothers know what they should do. (Group 7, female, participant ID number: G7F1)

Many participants shared their practices of teaching children about water safety, emphasizing avoiding natural water bodies in the rainy season and deeper pools/ponds. They also emphasized the importance of parental supervision to prevent children from entering water bodies unsupervised.

I tell them [the children] not to around water bodies during the rainy season, because the water will rise, and we might be carried away by the water flow. I also don’t allow them to go the beach with their friends alone. If they want to go to water bodies, they must go with us, their parents. (Group 7, female, participant ID number: G7F1)

Many participants noted that news reports, including those on social media, about drowning incidents, prompted action to improve parental supervision of children around water.

Interestingly, many participants highlighted how news reports, including those shared on social media platforms, regarding drowning incidents, has prompted action on improving parental supervision of children around water.

Yes, the news from news broadcast and social media [influence my level of awareness on drowning]. These days, we get more cautious about taking our children to swim. I’m also very worried about having my children being supervised by anyone else. Especially children around the age of 5 years old. (Group 6, male, participant ID number: G6M1)

### Theme 3—Community Education and Skills Development

Participants discussed the need for specific skills training to prevent child drowning, emphasizing the importance of children learning to swim. All stated that public schools, where their children attended, provided swimming lessons as part of the Sports/Physical Education class. However, there was variation in the provision of lessons, with some children only receiving them in junior high school and others in both primary and junior high school. Participants then commented that the infrequency of these school-provided lessons hindered their children’s ability to swim proficiently. Participants reported that only a single swimming lesson at a public pool was conducted once every school semester, equating to one lesson every six months. Consequently, participants felt parents could not rely solely on these lessons and needed to teach their children themselves. However, many noted that most parents in the community could not swim, making it challenging to teach their children.

Swimming lessons are only provided by the school once per semester. That is why there’s no way the children can swim, except if their own parents frequently can take them to the swimming pool. . . . [But] the parents themselves here don’t know how to swim. [For the school-provided lessons] we need to pay for the swimming pool [entrance fee] and the transport [cost] and the pocket money. Around 50 thousand rupiahs [AUD 5] every time they go swimming, so it’s a lot. The swimming lessons are for primary school and junior high school students. (Group 6, female, participant ID number: G6F1)

Many participants noted financial challenges related to transportation and pool entrance fees. Some proposed government-funded swimming lessons and local pool facilities to alleviate travel and cost burdens.

If the government makes a swimming pool, around here, and have the children to learn to swim, that would be good. (Group 1, male, participant ID number: G1M1)

Some participants suggested government-provided first aid training for parents, particularly mothers and community members, to respond to child drowning incidents. However, the potential roles of nongovernmental stakeholders, such as community or nongovernmental organizations (NGOs) and private entities, in providing support for swimming, rescue, and first aid training were not mentioned.

The local government, relevant government departments should provide education for us, on how to rescue children. Parents need to be informed that if their children are already in the water and have drowned, their lives could actually be saved. . . . So, first aid training, we need the education. (Group 6, male, participant ID number: G6M1)

### Theme 4—Community Action

Participants shared their perspectives and experiences on community actions to enhance child safety, suggesting measures such as covering water bodies and creating safe areas for children away from water.

Some participants mentioned the need for community playgrounds, noting that children often play near water due to limited safe open spaces. However, participants did not mention the need for playgrounds to supervised or fenced.

I think [the community needs] a playground. A playground for the children, because we don’t have open space here, so children tend to play in the creeks or ditches. (Group 1, male, participant ID number: G1M3)

Some participants emphasized the need of establishing daycare and preschool centers in the community to mitigate child drowning risk. Participants suggested that childcare centers could provide supervision for primary school-age children, while older children were seen as capable of self-care. No participants mentioned the need for pre-school children to be supervised in childcare centers.

Yes, younger children could be supervised in daycare here, around the age of primary school. Junior high students can take care of themselves already. We’re worried about those of primary school age. They only care about the fun in playing in the water. (Group 2, female, participant ID number: G2F1)

Participants stressed the importance of installing barriers around wells, ditches, streams, and ponds in the community to limit children’s access to these water bodies. Some mentioned proactively covering wells to prevent accidents and noted incidents of people falling into wells, particularly children drawn by curiosity, as cues for action.

Ditches and streams can perhaps be covered [to reduce the risk of drowning]. . . . Such as with fences from bamboo, so that kids can’t climb over it. (Group 3, female, participant ID number: G3F2)

### Theme 5—Policy Development and Enforcement

Participants discussed the need for community-level policies and regulations promoting water safety. The importance of various policies was highlighted, including restricting unaccompanied children’s access to public bathing pools, monitoring water levels to inform residents about flood risks, and enforcing safe boating regulations and safety features including lifeguard presence on public waters. Some believed the government’s limited capacity hindered effective enforcement. While participants recognized multisectoral responsibilities, most showed limited understanding on specific sectoral responsibilities for prevention.

Streams or ponds, they need to have safety features. . . . The water level needs to be monitored, not to be over their capacity and flood. . . . In public places such as swimming pools, beaches, lifeguards must be present. These days, it is very rare for us to see lifeguards. No lifeguards, in fact. Perhaps the government has limited funding. Perhaps the SAR [Search and Rescue] team could help. But they [Search and Rescue team] only arrive once a drowning event has taken place. (Group 6, male, participant ID number: G6M1)

## Discussion

Child drowning is a leading cause of death among Indonesian children, especially in eastern provinces like WNT (Cenderadewi et al., 2024); however, child drowning prevention remain insufficiently understood in Indonesia (Cenderadewi et al., 2023). This study provides insights into community perspectives and practices on current protective strategies used to reduce child drowning risk and necessary preventive measures within the local context.

This study revealed a consensus among participants on the importance of individual-focused interventions for preventing child drowning in Indonesia. Analyzing community perspectives and actions using the HPF (Talbot & Verrinder, 2017), it was revealed that participants sought educational interventions, particularly swimming lessons for school-age children. This aligns with findings from similar LMIC settings, such as the SwimSafe Program in Bangladesh, which demonstrated the effectiveness of survival swimming skills in averting drowning incidences and fatalities (Rahman et al., 2012). Furthermore, participants noted that many parents in the community could not swim, which hindered both teaching children to swim and providing immediate assistance during drowning emergencies. This gap highlights the need for targeted training programs to also equip parents with swimming and water rescue skills, enabling them to respond effectively in such situations.

Participants recognized the importance of life-saving skills, particularly for mothers, although with less clear distinctions between water rescue and first aid. This aligns with WHO’s recommendation to equip parents and community leaders with rescue and resuscitation skills (WHO, 2014, 2017, 2022) Studies in similar LMIC settings, such as a first responder training program in Bangladesh, demonstrate that communities in low-literacy environments can successfully acquire life-saving skills through hands-on training and low-literacy education materials, such as videos and posters (Rahman et al., 2014). Further research is needed to determine effective methods of equipping Indonesian parents and community members with safe water rescue and resuscitation skills, by exploring context-specific adaptations tailored for communities in rural, low-literacy settings.

Participants also acknowledged the importance of both midstream and upstream interventions for drowning prevention. Recognized midstream interventions included establishing safety measures around water bodies and community safe places for children. Upstream interventions included advocating for policies to limit access to water bodies, safety compliance, disaster hazard identification, and intersectoral collaboration across government sectors. This recognition of midstream and upstream interventions aligns with the tenets of the HPF, which advocate for a continuum of approaches, encompassing educational, behavioral, socio-environmental, and regulatory measures (Franklin et al., 2012; Franklin & Sleet, 2018; Leavy et al., 2016; Scarr & Jagnoor, 2022). These findings also resonate with the WHO’s recommendations on the need for cross-sectoral partnerships for sustainable, nationwide drowning prevention efforts (WHO, 2017, 2022)

Although many participants acknowledged the importance of interagency collaboration for effective drowning prevention, most had limited understanding of different government departments’ responsibilities and objectives. Specifically, there was an awareness gap on the crucial roles of the education and health departments in child drowning prevention, despite being recognized as key actors in many countries’ multi-strategic approaches to drowning (Leavy et al., 2016; Scarr & Jagnoor, 2022; Szpilman et al., 2014; Wallis et al., 2015). In this study, only one participant explicitly mentioned the education sector’s responsibility to formally integrate swimming lessons into school curriculum. Although participants reported some level of swimming lessons were available in schools, implementation was inconsistent, and formal integration into the curriculum was lacking.

Participants lacked an understanding about how responsibilities are coordinated or divided between national and local government levels, highlighting the need for improved understanding and clarity regarding how different government levels collaborate to address water safety. Given Indonesia’s archipelagic nature and large population and the multifaceted dimension of drowning, there is a pressing demand for systematic integration of drowning prevention frameworks across regulatory activities, spanning water safety enforcement, child protection, childcare, education, health services, safety infrastructure, boating regulation, disaster management, and addressing economic disparities (Cenderadewi et al., 2023). Clear delineation of responsibilities and coordination mechanisms among agencies is crucial for cohesive national prevention efforts (Cenderadewi et al., 2023).

Participants demonstrated limited awareness of nongovernmental stakeholders’ relevance to drowning prevention, despite past research highlighting their global contributions (Jagnoor et al., 2021; Scarr, 2014; Scarr & Jagnoor, 2022). A previous review indicated non-health sectors often lead drowning prevention efforts in many countries, with increasing NGOs involvement in policy, implementation, and research, offering opportunities for aligning child drowning prevention with broader health and development goals (Scarr & Jagnoor, 2022). As recommended by the WHO, fostering multisectoral collaboration should begin by identifying and connecting with all relevant collaborators, including industry, academia, civil society, NGOs, international bodies, and local communities (WHO, 2017). Further research is crucial to engage these stakeholders effectively in Indonesia’s prevention efforts at all levels.

Participants recognized the importance of both face-to-face and media communication to raise awareness about drowning risks and prevention. This aligns with WHO’s recommendation to improve public awareness through strategic communication (WHO, 2014, 2017, 2021) as well as with the HBM construct, which asserts that individuals are unlikely to adopt preventive behaviors without perceiving susceptibility (Champion & Skinner, 2008). This underlines the need for awareness-raising initiatives, as a precursor to the adoption of safety behaviors, to be integrated into a strategic drowning prevention plan (WHO, 2014, 2017, 2021)

While this study provides valuable insights into how parents and community members perceived child drowning prevention, further research is imperative for developing contextually relevant water safety promotion approaches and the alignment of cross-sector partnerships in Indonesia. Investigating social determinants of drowning within the Indonesian context, is vital to ensure the effective and sustainable adoption of life-saving practices within underserved and culturally diverse communities across Indonesia.

Despite the strengths of the study, a number of limitations should be acknowledged. While the sampling method ensured geographical diversity between coastal areas and areas around inland water bodies, the study focused solely on one specific region of Indonesia. Considering Indonesia’s extensive geographical and cultural diversity, further research is needed nationwide to determine whether perceptions and actions related to child drowning prevention are consistent. In addition, participants were self-selected and might diverge from the broader community’s perspectives.

The limited participation of fathers was reflective of the cultural norms in the study setting, where childcare responsibilities and discussions about child safety are typically considered the domain of mothers. As a result, snowball sampling referrals frequently identified mothers as potential participants, and more mothers accepted the invitation to participate. Furthermore, fathers’ work commitments further hindered their participation despite scheduling accommodations. Moreover, the FGD composition may have limited comparisons between parents’ and community leaders’ perspectives.

## Conclusion

This study highlighted parents and community members’ focus on individual-focused, interventions for drowning prevention, particularly on educational programs on swimming skills for school-age children and life-saving skills for parents and community members. There was limited understanding of the roles of education and health departments in child drowning prevention efforts. Although participants reported some level of swimming lessons were available in schools, implementation was inconsistent, and formal integration into the curriculum was lacking. Further research into formally integrating swimming training into school curricula and assessing its impact on reducing child drowning rates, developing contextually relevant water safety promotion approaches, and fostering cross-sector partnerships are crucial steps to ensure effective and sustainable drowning prevention efforts in Indonesia.

## Appendix

**Appendix A.** Focus Group Moderator Guide’s Domains of Enquiry and Examples of Follow-Up Questions and Probes.

Domains of enquiry and examples of follow up questions and probes	Constructs of Health Belief Model applied
• **Q1. “Could you tell me some of the activities your family engaged with around water bodies?”** - Family’s relationship with water: • “Could you tell me about your family’s and children’s activities around water?” • “Could you tell me what water bodies exist in your community and in and around your home?” • “Could you tell me your use of watercrafts and flotation devices on board?” - Supervision for children: • “Could you tell me more about your family structure and the main caretaker in your household?” • “Could you tell me who in your family is responsible to supervise children while they are doing activities around water?”	• Perceived susceptibility
• **Q2. “What do you think are the greatest health concerns for your community?”** - “How important do you think drowning is as a health issue in your community?” - “Where do you think drowning fits among these greatest health concerns in your community?” - “Have you ever experienced/witnessed/heard stories about drowning events in your community?” - “Are you aware of local beliefs and practices surrounding the issue of drowning in your community?”	• Perceived susceptibility • Perceived severity • Cues to action
• **Q3. “Who do you think is at most risk for drowning in your community?”** • **Q4. “What do you think of drowning as a cause of injury/death for children?”** • **Q5. “What do you think are the reasons that might cause a child to drown?”** • **Q6. “Can you tell me about aspects of the environment and community in which you live that could increase the risk of a child drowning?”**	• Perceived susceptibility • Perceived severity • Perceived barriers • Self-efficacy • Cues to action
• **Q7. “What are some of the things that might make it hard to keep children safe from drowning?”** • **Q8. “Can you tell me about how and what have you taught your children about water dangers?”** • **Q9. “What would you like to see put in place to prevent children from drowning in your community?”** - “Who do you think are responsible for preventing drowning?” - “Where do you get your information on drowning prevention from?” - “What are some of the things that might make it more difficult for you to apply this in your community”?	• Perceived barriers • Perceived benefits • Self-efficacy • Cues to action

**Appendix B.** Focus Group Participants’ Characteristics and Home Environment.

Focus group participants’ characteristics and home environment (*n* = 62)		*n*	**%**
Gender	Female	47	75.8
	Male	15	24.2
Age group	18–24 years	6	9.7
	25–34 years	16	25.8
	35–45 years	16	25.8
	44–54 years	11	17.7
	55 and above	13	21.0
Type of participant	Mothers	33	53.2
	Fathers	6	9.7
	Village community leaders	23	37.1
Education level	Did not complete primary education	36	58.1
	Completed primary education	14	22.6
	Completed high school	12	19.4
Occupation	Homemaker	34	54.8
	Daily laborer	7	11.3
	Farmer	4	6.5
	Fisherman	2	3.2
	Others	15	24.2
Number of children	0	1	1.6
	1	10	16.1
	2	11	17.7
	3	16	25.8
	4	12	19.4
	5 and above	12	19.4
Number of children below the age of five	0	23	37.1
	1	28	45.2
	2	11	17.7
Water bodies exist within 500 meters from participant’s home	Wells	62	100.0
	Ditches	46	74.2
	Creeks	46	74.2
	Rivers/larger streams	13	21.0
	Beaches	12	19.4
	Ponds	1	1.6
Water bodies exist in and around participant’s dwelling	Wells	60	96.8
	Bathtubs (serves as water containers)	50	80.7
	Bucket	55	88.7
